# A realist evaluation protocol: assessing the effectiveness of a rapid response team model for mental state deterioration in acute hospitals

**DOI:** 10.3389/frhs.2024.1400060

**Published:** 2024-07-15

**Authors:** Tendayi Bruce Dziruni, Alison M. Hutchinson, Sandra Keppich-Arnold, Tracey Bucknall

**Affiliations:** ^1^ School of Nursing and Midwifery, Deakin University, Geelong, VIC, Australia; ^2^ Alfred Health, Melbourne, VIC, Australia; ^3^ Centre for Quality and Patient Safety Research, Institute for Health Transformation, Deakin University, Geelong, VIC, Australia; ^4^ Barwon Health, Geelong, VIC, Australia

**Keywords:** realist evaluation, mental state deterioration, clinical risk management, clinical aggression, clinical decision making, restrictive interventions

## Abstract

**Background:**

Mental state deterioration poses significant challenges in healthcare, impacting patients and providers. Symptoms like confusion and agitation can lead to prolonged hospital stays, increased costs, and the use of restrictive interventions. Despite its prevalence, there's a lack of consensus on effective practices for managing mental state deterioration in acute hospital settings. To address this gap, a rapid response team model has been proposed as a potential intervention, aiming to provide early identification and targeted interventions.

**Methods:**

Based on realist evaluation steps, first, initial program theories are formulated to understand the logic behind the intervention. Second, literature synthesis identifies empirical evidence on contexts, mechanisms, and outcomes elements, refining initial theories. During the third step, data will be collected using qualitative methods such as field observations and interviews, as well as quantitative methods such as surveys of the staff, audits of electronic medical records, and analysis of incident records of mental state deterioration. Analysing this data informs configurations of contexts, mechanisms, and outcomes. In the fifth step, the configurations are synthesised, presenting refined, evidence-informed program theories.

**Conclusion:**

This study addresses the knowledge gap by evaluating the rapid response model's effectiveness in managing mental state deterioration in acute hospital settings. Realist principles guide the exploration of causal mechanisms and their interaction with specific implementation contexts. The objective is to identify what works, for whom, and under what circumstances, aiming to manage deterioration, reduce restrictive interventions, and enhance the experience for patients and staff by implementing a proactive model of care. The findings contribute to evidence-based approaches for managing mental state deterioration in hospital settings, informing policy and practice in this crucial area of healthcare.

## Background

Managing mental state deterioration (MSD) presents numerous challenges, and the available evidence highlights the need for organisations to prioritise the implementation of interventions to improve the quality of patient care (1–4). To date, limited research has been conducted on the effectiveness of systematic approaches that recognise, escalate, manage, and report MSD in healthcare settings (5, 6). MSD can occur across all healthcare environments and an acute deterioration in a person's mental state is a negative outcome by itself and may lead to additional serious consequences, such as suicide, aggression, and prolonged hospital stays (1).

The impacts of patient MSD include the use of restrictive interventions, challenges in managing unpredictability, risk of injuries, impact on staff morale, and poor job satisfaction (3). Restrictive interventions include seclusion, defined as confining someone to a space they cannot leave; bodily restraint, comprising physical and mechanical means to limit movement, where physical restraint excludes minimal necessary support for daily activities or guidance for the disoriented; and chemical restraint, which involves using medications to control a person's behaviour by restricting their freedom of movement, excluding their use for treatment purposes (7). Evidence indicates that the continued use of restrictive interventions such as restraints, which are within national guidelines (7, 8), may further traumatise patients and may result in negative outcomes such as longer hospital stays (9, 10).

Mental state deterioration refers to the decline or worsening of a person's mental or psychological well-being, which might indicate the need for additional care (11). Mental state consists of mood, behaviour, orientation, judgment, memory, problem-solving ability, and contact with reality (12). Indicators of deterioration may include reported change in mood, sleep disturbances, loss of touch with reality, loss of ability to adhere to medical treatments, and elevated risks to self, others such as expressions of suicidal thoughts and self-harm (13, 14). As a result, MSD can negatively impact an individual's quality of life, functioning, and overall health outcomes.

The factors that contribute to patient MSD are multifactorial. In acute hospital settings, they may include medical interventions such as surgical procedures which can impact mental health; the use of anesthesia, the physical stress of surgery, and postoperative pain are potential triggers for MSD. Prolonged isolation, especially in medical settings or during restrictive visitation policies can exacerbate feelings of loneliness and helplessness when patients do not have regular contact with family and friends, leading to significant emotional distress. Factors may also include personal characteristics such as deterioration of mental health conditions, cognitive impairment, physical health conditions, including delirium, atypical reactions to prescribed treatments, or inebriation from illegal substances (15, 16). Furthermore, patients can also experience deterioration because of variables emerging from their social settings or in response to the environment, making anticipating indications of deterioration a complex task (1, 6).

Internationally and in Australia, managing patients presenting with MSD has been recognised as an important issue for healthcare workers and a workforce concern for organisations (2, 17, 18). For burnout, symptoms experienced by healthcare staff include varied degrees of emotional exhaustion and low perceived professional efficacy, which are linked to poor occupational satisfaction and performance (13, 18). For organisations, the impacts include financial costs associated with sick leave, legal liabilities, providing substandard clinical care, and challenges with retaining and recruiting staff (19–21).

A meta-analysis by Y-L Li, R-Q Li, D Qiu and S-Y Xiao (18) found that about 62% of participants from 253 international studies reported exposure to workplace violence. Healthcare workers are at a greater risk of exposure to aggression when caring for patients presenting with MSD than in other industries (22, 23). The Royal College of Nursing (24) in the United Kingdom reported that about 70% of the staff across all hospital settings cared for patients presenting with MSD. Researchers, healthcare professionals, and decision-makers globally have increasingly recognised the significance of addressing and managing MSD among patients.

Researchers at a Melbourne tertial hospital found that aggression was the most important concern for staff working in mental health settings (25). Previous research examining aggression among patients in acute mental health settings led to the development of tools such as the Dynamic Appraisal of Situation Aggression (DASA) introduced as part of the risk assessment and management process rather than using unstructured clinical judgement alone (26). The DASA is a 7-item scale that aims to assist in the short-term (24-h) assessment of patients with an increased risk of violence in mental health settings (27). The DASA, endorsed by the National Institute of Clinical Excellence in the United Kingdom as an actuarial prediction instrument, is used in Finnish and French psychiatric services, and has been trialled by a forensic mental health service in Melbourne, Australia (4, 10, 26, 28).

Currently, interventions in acute medical settings are often triggered after visible signs of MSD are evident through Code Grey, Code Black, and referral processes to mental health teams which takes time, contributing to delayed responses and resource-intensive crisis management. Consequently, current approaches are reactive and inconsistent, rather than proactive, likely contributing to high rates of MSD in acute hospital settings (11, 17). Code Grey is an organisation-level response to genuine or possibly violent, aggressive, harmful, or threatening conduct shown by patients or guests towards others, creating a health and safety challenge (29). Code Black is a hospital's internal security response to potential and actual behaviour involving a weapon or severe threat to personal security (30).

Code Grey standards (Governance, Personnel, Procedures, Outcomes, and other resources) were released in 2017 to facilitate consistent practice in responding to occupational violence and aggression in Victorian public healthcare services in Australia (29). An analysis of Code Grey responses found that responses were associated with primary diagnoses and mental illness problems, alcohol and drug addiction, disability, personality disorder, acquired brain injury, delirium, and altered cognition (25). However, Arnetz et al. (5) found a lack of empirical evidence supporting the implementation of system-wide approaches to patient MSD prevention and management. There is a considerable body of research relating to the management of clinical aggression in settings such as emergency departments, mental health settings, and geriatric care (3, 31, 32).

Despite the efforts highlighted above, researchers from a Melbourne tertiary hospital found that staff felt unsafe, unsupported, and often under-skilled to manage patients presenting with MSD in mental health settings (33). Moreover, the literature shows a lack of systematic approaches to identify, recognise, document, and escalate the early warning signs of MSD, and the incidence rates remain high (17). To address this knowledge gap, a tertiary hospital in Melbourne will trial the DIvERT (De-scalation, Intervention, Early, Response, Team) intervention to address patient MSD in acute hospital settings. In Table 1, the DIvERT Intervention Black Box illustrates what the model involves, what resources are provided, and the predicted outcomes from its implementation. We believe that between the intervention resources and the intended outcomes is the “Black Box” of the mechanisms DIvERT is intended to activate, all embedded in layers of underlying context.

**Table 1:** DIvERT intervention black box.

What DIvERT involves
• The aim of DIvERT is to initiate early intervention at first signs of patient mental state deterioration and mobilise a timely response to manage MSD. • The early intervention aims to ensure a timely, appropriate care plan to support the team to proactively manage patient MSD risks from escalating. • DIvERT will form a three-level escalation response for patient MSD, including the local response, the DIvERT response, the Code Grey, or Code Black. • The DIvERT team configuration will likely comprise include the bedside nurse, Associate Nurse Unit Manager, home team registrar, Clinical Liaison Psychiatric, Allied Health staff such as Social Worker, and Psychologist
DIvERT intervention resources
• Training programs for medical ward staff to improve their skills in recognising and escalating the signs of MSD. • Efficient communication systems to facilitate timely and clear information exchange among healthcare team members, ensuring a rapid response to MSD risks. • Clearly defined protocols and guidelines outlining the steps to take in the event of MDS, ensuring a standardised, effective, and timely response. • Validated tools for assessing the risks of MSD, supporting the assessment of risks that may require timely intervention. • Individualised intervention plans for patients identified as at risk, outlining specific strategies and interventions tailored to their needs. • Access to appropriate medications and mechanisms for their timely administration when recommended by the response team. • Systems for collecting and monitoring data for MSD incidents, allowing for ongoing evaluation and improvement of DIvERT. • Established procedures for follow-up to support patients after MSD presentation, promoting continuity of care and preventing further deterioration. • Training programs to ensure staff have the skills to confidently communicate with patients using trauma-informed care approaches and are knowledgeable about the intersection vis a vis physical health and mental health. • Training programs to ensure cultural competence among staff, recognising and respecting the diversity of the population.
Intended outcomes
• Improved model of care for patients presenting with MSD in medical settings. • Improved mental health outcomes for patients in medical settings, including integrated care, timely intervention and treatment, reduced hospital admissions, and improved overall mental well-being for patients. • Implementation of standardised protocols for managing MSD, ensuring consistency and effectiveness approaches through DIvERT. • Reduced incidence of Code Grey and Code Black. • Increased safety for both patients and healthcare staff by addressing potential risks associated with MSD in a timely and effective effort. • DIvERT will present an opportunity to trial an evidence-based model of care for managing MSD for other wards to implement. This is done by refining the DIvERT program theory given contextual factors. • Improved patient and family experience of high-quality hospital care. • Build staff capability and confidence in recognising and managing MSD. • Opportunities for continuous quality improvement through ongoing evaluation and refinement of DIvERT's protocols and interventions. • Develop a cultural change shift and nursing empowerment for managing mental state deterioration. • Positive effects on the well-being of healthcare staff, as they will feel better equipped and supported in managing challenges, potentially reducing stress and burnout associated with managing MSD.

DIvERT, De-escalation Intervention Early Response Team.

## Aim

Based on realist evaluation methodology, this study is designed to evaluate the effective functioning of the DIvERT program, an early intervention designed to respond to and manage a mental state deterioration in patients in selected acute hospital settings. The primary objective of the study is to provide a causal explanation of how the DIvERT program works, for whom it is effective, and under what specific circumstances it leads to positive outcomes. By exploring these key aspects, the aim of the study is to contribute to understanding the program's mechanisms and inform its implementation in clinical practice.

## Objective

In accordance with the realist evaluation methodology, the objectives of this research are:

1. Theory articulation: Hypothesise the DIvERT program purpose by theorising, classifying and organising the assumptions of the program designers and implementers into initial program theories.
2. Theory refinement: Search, review and synthesise the literature for existing empirical evidence on what works, for whom, under what circumstances, and to what extent when managing mental state deterioration in patients in acute general hospital settings.
3. Theory verification: Analyse evidence to determine the contexts, mechanisms of action, and outcomes of the DIvERT program.
4. Theory refinement: Present refined program theories with the corresponding context, mechanism, and outcome configurations to explain the DIvERT program and account for what did not work.
5. Interpretive meeting with key stakeholders at the conclusion of a realist evaluation to validate findings, refine program theories, identify unintended consequences, gather contextual insights, provide practical recommendations, and improve the credibility of evaluation.

## Method

### DIvERT intervention

**Figure 1:**
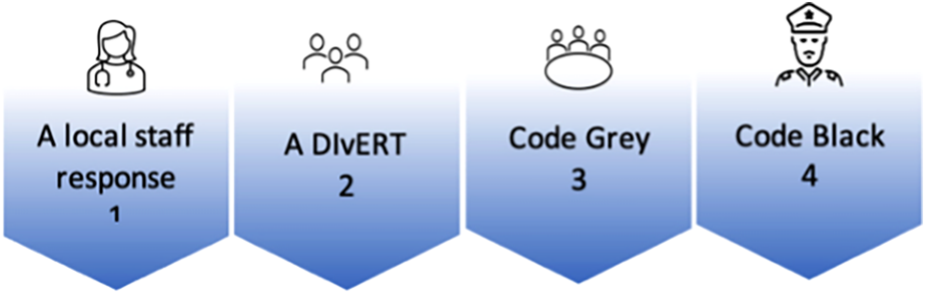
Four levels of escalating mental state deterioration.

The DIvERT is designed for health professionals to intervene at the first sign of MSD and mobilise a timely response to manage the patient's MSD. This early intervention strategy is intended to ensure that the patient receives an appropriate and timely care plan and for the team to manage deterioration and prevent associated risks from escalating. DIvERT will be piloted in two selected clinical settings of an Academic Health Science Centre of a teaching hospital: a trauma unit and an acute surgical ward specialising in burn services; plastics; reconstructive surgery; and ear, nose, and throat surgery. DIvERT was commenced end of 2019 however, due COVID the pilot was stopped. The trial recommenced in 2021. Following implementation, if successful, DIvERT will form part of the hospital escalation response process for MSD, as illustrated in Figure 1.

The first response involves ward staff responding and attempting to manage the patient's deterioration. DIvERT is a second level response utilised when the patient's condition and symptoms become unmanageable for the ward staff. DIvERT involves a multidisciplinary team that provides timely and targeted interventions, assists with patient management, and makes recommendations to the ward team to address the patient's needs. The third level of escalation and response is Code Grey and the fourth level is Code Black.

### Realist evaluation

Realist evaluation is a theory-driven evaluation methodology rooted in a realist philosophy of science, which emphasises an understanding of generative causation and how causal mechanisms are shaped and constrained by context (34). Generative causation refers to the idea that underlying mechanisms (causal forces) are triggered in particular contexts, which may not always be observable, and generate or lead to outcomes (35). The realist methodology is well suited to complex interventions that require multi-level organisational changes and involve human decisions and actions. The realist approach is intended to answer the questions, “what works, for whom, under what circumstances and how” instead of “does it work” (36, 37). In order to achieve this, it is necessary to uncover the architecture of programs, services, formal and informal resources, and efforts (38). R Pawson (39) argues that programs are incarnate theories; therefore, an implemented program is constantly testing theories about what might lead to changes.

The realist philosophical paradigm, realism, positions itself between the positivist and constructivist paradigms. Positivism primarily aligns with the hypothetico-deductive method to verify *a priori* hypotheses using quantitative approaches, wherein functional relationships are determined between causal and explanatory factors (40). Conversely, constructivism is aligned with qualitative research approaches, which emphasise the fact that reality is constructed through human interaction and action (41). Realism, thus, is the “middle ground”, which is the search for the alignment between reality and our constructions of reality (42). The realist ontology holds that reality exists independently of our perception of it and can, therefore, neither be observed nor perceived (43). Therefore, the realist understanding of the world recognises it as an open, complex, and nested system with different levels of stratified reality, the real domain, actual domain, and empirical domain (42, 43). Real refers to structures with causal powers and liabilities that enable or constrain actions, the actual refers to events or non-events that are enabled or constrained by the real domain, and the empirical refers to the observations and experiences that are a result of the actions and events in the actual domain (43). As well as acknowledging the social construction of reality, realists acknowledge that reality stems from a complex interplay between social, geographic, and historical context; hence, what is considered to be factual would likely change over time (39).

In realist methodology, we are interested in identifying and understanding people's responses to different resources offered within complex interventions (44). Rather than focusing on what can be seen at the surface level (empirical and actual domain), the realist approach is focused on understanding the complex layers that exist beneath the surface level (real domain) and explaining the reasoning behind human actions (42, 43). To do so, researchers must unpack the underlying generative mechanisms that explain “how” outcomes arise and the influence of context (35, 37). The realist driving concepts are retroduction, the search to unearth causal mechanisms (underpinning causal forces), and ontological depth. Thus, reality is stratified, the observable is a surface layer, and the causal features transpire at a deeper level (38, 45).

A significant advantage of a realist evaluation in enhancing evidence-based practice is its emphasis on detailed contextual analysis to provide explanatory insights into how a program functions (37). Furthermore, realist evaluations provide a valuable framework for conceptualising the underlying theories of a program. By examining and identifying the Context, Mechanism, and Outcome (CMO) configurations, researchers can better understand the interplay between contextual factors, mechanisms, and the resulting outcomes (intended and unintended). This is generally summarised as context + mechanism = outcome (42). The causal explanation resulting from this analysis will help the construction of a deeper understanding of why specific regularities occur and how they contribute to program outcomes. Table 2 provides a definition of context, mechanisms, and outcomes.

**Table 2:** Definitions of context, mechanism, and outcomes (42).

Context Contexts encompass the relevant features of the conditions in which programs are implemented, which are essential for understanding the functioning of mechanisms. They represent the elements in the background environment that exert an influence on program outcomes
Mechanism Mechanisms explain what it is about programs and interventions that bring about changes. It is not programs that work but the resources they offer that enable users to make them work. Fundamentally, mechanisms are the engines of explanation in realist evaluation
Outcome Also known as outcome patterns, outcomes comprise the intended and unintended interaction resulting from the activation of different mechanisms in different contexts. They can be proximal, intermediate, and distal

This evaluation will be reported in line with the RAMESES (Realist And Meta-narrative Evidence Syntheses: Evolving Standards) reporting standards for realist evaluations (46). Accordingly, we will follow the realist evaluation procedural steps as illustrated in Table 3.

**Table 3:** Realist evaluation steps, adapted from R pawson and N tilley (42) and FC mukumbang, B marchal, S Van belle and B Van Wyk (47).

Step 1	Construct initial program theories i.e., use practical expertise, literature, stakeholder consultation, and retroductive thinking. Theories are not only descriptive, but they also attempt to describe causal relationships
Step 2	Develop research protocol based on theories i.e., plan for database searching, screening, and retaining literature, appraisal, plan for who to interview, and what to ask based on initial program theories
Step 3	Gather evidence based on initial theories i.e., execute plans with an eye for iteration and modification. Mixed methods approach involving in-depth interviews and non-participant field observations
Step 4	Configure collected data about initial theories i.e., organise and analyse data using CMO configurations, triangulation of CMO data using ‘causal loop thinking” to map the bigger picture, refining CMO configurations into program theories
Step 5	Present the research output i.e., CMO configurations and evidence-informed theory to address “what works, for whom, under what circumstances and how?”

### Step 1: construction of initial program theories

Initial program theories (IPT) are a fundamental prerequisite of the evaluation methodology. The essential concept is, “If we do x, y will happen, because…,”. The “because” is critical to the structure; without it, it would just be a theory of action (48). “If… then” statements are a way to begin theorising, classifying, and organising the experiences and assumptions of the program designers and can lead to hypothetical CMO configurations (38, 49). IPTs can remain as “if…. then” statements, which become unpacked in the data analysis using CMO configurations. It has been suggested that CMOs can be developed concurrently either during the IPT development (for example, during the initial round of an evaluation, for testing during later stages) or retrospectively as a product of the evaluation rather than a design tool (48). As the most fundamental claims about an intervention, theories are seen as the starting and end point of the realist evaluation cycle (37).

IPTs are outlined in abstract terms and are concerned with identifying and explaining regularities (42). As pointed out, interventions are theories put into practice (50). Developing theories involves determining the logic of an intervention, deriving underlying assumptions about the effectiveness of the intervention, and explaining how it is expected to work. To construct our theories on a rapid response team model for managing MSD in patients, a comprehensive search plan will be developed, and a thorough scoping of published research and grey literature will be conducted. This approach ensures that a wide range of relevant information and evidence is gathered to inform the construction of initial theories. Incorporating published research as well as grey literature allows us to capture diverse perspectives and valuable insights that are not readily available in traditional academic publications. The search plan and scope will allow us to consider a variety of sources and contribute to a comprehensive understanding of the rapid response team model in managing patient MSD. In addition to conducting a literature review to gather relevant information, the process will be supplemented with creative brainstorming to develop the best explanation for how DIvERT is intended to work. As part of the second step of the realist evaluation cycle, initial theories will be tested and refined through realist synthesis (51).

### Step 2: search for literature evidence to test initial program theories

**Figure 2:**
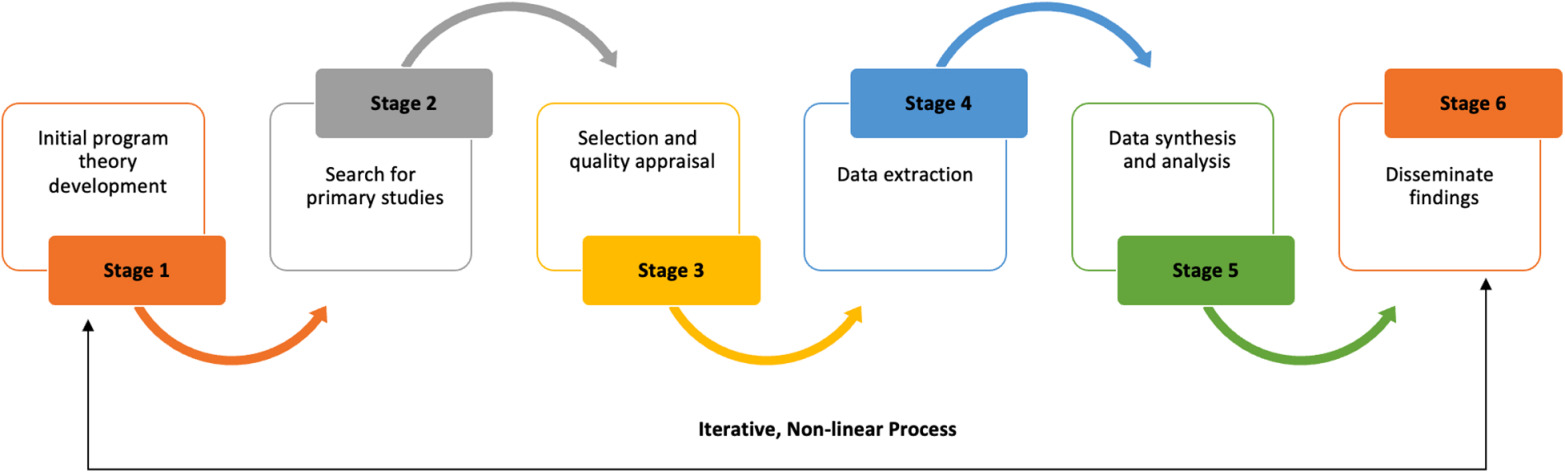
Realist synthesis steps.

In Step 2, we will complete a realist synthesis of relevant literature to test, refute, and refine our IPT. The synthesis will be and reported in line with the RAMESES (Realist And Meta-narrative Evidence Syntheses: Evolving Standards) publication standards for the realist synthesis (51). A realist synthesis is a theory-driven approach to synthesise the literature for empirical evidence to generate insights into complex interventions or programs and how they work in specific contexts (52). In contrast to traditional systematic reviews, realist synthesis attempts to gain a deeper understanding of the underlying mechanisms, contextual factors, and interactions that shape outcomes (51). Realist synthesis aim to answer not only the questions of “what works” but also “how, for whom, and under what circumstances” (42). Based on the realist synthesis interpretative, theory-driven approach, we will iteratively search for qualitative, quantitative, and descriptive literature to understand and unpack the underlying generative mechanisms (M) that explain “how” outcomes (O) are caused and the influence of context (C). We will follow the methodological steps outlined in Figure 2 to complete and report our synthesis based on RAMESES publication standards (51).

Refined program theories are intended to highlight the underlying generative mechanisms that contribute to the effectiveness of the rapid response team model in effectively addressing MSD. In addition, theories will consider the contextual factors that influence the implementation and effectiveness of the response model (53). The refined program theories resulting from the synthesis will be shared with the program designers within the organisation as part of the theory gleaning process. This collaborative step involves engaging with the program designers to examine and evaluate the refined theories to determine and prioritise theories for further testing in the next stage of the evaluation (51, 53). Program designers will review and provide input on the refined program theories during the theory gleaning process. Based on their expertise and knowledge of the intervention, they will evaluate whether theories are aligned with their initial assumptions and expectations (54). The objective is to gain deeper insights from program designer perspectives and determine which theories are most plausible and worthy of further testing. Through this collaborative engagement, the program designers can contribute valuable insights and identify potential additional contextual factors, mechanisms, or outcomes (53, 54). Discussions and feedback gained from the theory gleaning process will assist in refining the theories further, identifying additional contextual factors, mechanisms, and outcomes, and planning the design of data collection methodologies for the next phase (52, 54). Refined program theories are intended to highlight the underlying generative mechanisms that contribute to the effectiveness of the rapid response team model in effectively addressing MSD. In addition, theories will consider the contextual factors that influence the implementation and effectiveness of the response model (53).

### Step 3: gather evidence based on initial theories

As part of the realist evaluation methodology, researchers are guided by initial program theories (IPT) when selecting the appropriate instruments and measurement tools (48). As a basis for testing and refining the program theories, IPT's serve as a framework for identifying and collecting relevant data, such as interviews, observations, surveys, and document analyses (46, 55). The realist approach offers the advantage of integrating the strengths of both positivist and constructivist research paradigms. The positivist approach facilitates the collection of empirical evidence on program effectiveness, identifying patterns and quantifying the relationships between inputs, processes, and outcomes (34, 40). On the other hand, the constructivist approach provides rich contextual information, examines the perspectives and experiences of individuals and groups, and uncovers the underlying mechanisms and causal processes (34, 41). By embracing the complexity inherent in healthcare research, the realist methodology offers a sophisticated and nuanced understanding of research problems and questions, leading to comprehensive and insightful findings (56).

### Quantitative data collection

The DIvERT (De-scalation, Intervention, Early, Response, Team) intervention will be trialled in specific clinical settings within a tertiary teaching hospital in Melbourne, Australia. The clinical settings offer unique contexts that encompass differences in patient characteristics, presentations of MSD, and staff's experiences in managing patient deterioration. As part of the healthcare organisation's MSD monitoring and reporting process, electronic medical records, risk management systems, incident reporting systems, and security reporting systems are all used to collect MSD data. We will carry out a quantitative analysis of patient MSD data to gain a better understanding of the “what is going on” phenomenon by examining a range of variables, including but not limited to the patients' demographic characteristics, medical histories, factors contributing to MSD, how the MSD episodes were managed, and any interventions or treatments provided during the DIvERT responses. The analysis is also aimed to assess and establish the outcomes of patients who experience MSD, such stabilisation of MSD, length of stay in hospital, recurrent MSD episodes, and long-term management plans such as behavioural management plans for patients with complex MSD.

It is noted that, a current challenge faced by healthcare organisations is the lack of reliable systems for collecting quality data to inform continuous monitoring and improvement (17). Reporting MSD incidents serves as a foundation for promoting patient safety, facilitating data-driven decision-making such as supporting the implementation of interventions like DIvERT, advancing research efforts, ensuring regulatory compliance, and fostering a culture of continuous improvement in clinical practice (4, 57, 58). By complying with reporting requirements, healthcare systems establish accountability and promote transparency, ultimately enhancing the overall quality of care provided.

### Incident reporting systems

The organisation uses multiple incidents reporting databases, including Code Grey, DIvERT, electronic incident reporting forms, and security reports. These incident reports serve as the basis for testing different theories, such as theories relating to the prompt intervention, multidisciplinary collaboration, early warning signs recognition, tailored interventions, and clinical skills development. By analysing incident reports within these theoretical frameworks, we hope to gain insights into the effectiveness of DIvERT and understand how different factors, such as timing of interventions, collaboration between response team and ward staff, recognition of warning signs, and person centred approaches to managing MSD, contribute to the effectiveness of DIvERT.

Incident analysis is undertaken to uncover the key factors influencing outcomes, including effective and timely intervention preventing MSD risks from escalating to Code Grey, positive patient and staff experience, and positive organisational culture. These incident reporting systems capture comprehensive data, including demographic details, incident specifics, contributing factors, and immediate actions taken. Additionally, they are part of the organisation's risk management and clinical governance efforts, providing data on interventions, response outcomes, and follow-up plans for continuous improvement in patient and staff safety.

### Electronic medical records

We will use electronic medical records to collate data missing from DIvERT, Code Grey, and security data records. Electronic health records will provide valuable insights into how the response team addresses individual patient characteristics and needs in managing MSD and the outcomes of patients presenting with MSD. In addition, the records will provide insights into how the response team considers and manages social, environmental, and other factors that may affect the patient's mental state. The evaluation is also aimed to test theories related to shared decision-making and handover, including patient involvement, and the provision of resources to support staff in caring for patients following an episode of deterioration.

### Staff survey

We intend to survey clinical staff who work in the medical units where DIvERT is being trialled. The survey will enable the collection of self-reported data on variables such as training, confidence levels, and factors affecting communication, assessment, and documentation, providing insight into the perspectives and experiences of nurses. Additionally, we are interested in assessing staff ability to recognise early warning signs of patient MSD and understanding through free text responses, the contextual factors of what should be in place for DIvERT to be effective. Using the survey as a theory confirmatory approach, the objective is to empirically test, refine, and validate the underlying assumptions and causal mechanisms for evidence that best explains how an intervention works (34, 59). This ensures that the theories are grounded in empirical evidence and can inform effective decision-making and program improvement. The survey methodology is preferred for its systematic and efficient nature, which allows for the collection of valuable data from the participants (60).

Based on IPT's we will develop a survey instrument in line with the principles and criteria for designing survey questionnaires to test and refine our theories (61). Survey data will be collected and managed using REDCap electronic data capture tools hosted by the healthcare organisation. Additionally, we will provide a printed copy of the survey instrument for staff to complete to increase uptake. We will complete a pilot test of the survey instrument with select nurses to ensure consistency, clarity, and readability, as well as to check for technical issues, evaluate response options, assess question relevance, and ensure clear instructions.

### Data recording procedure and sample size

A Microsoft Excel spreadsheet will be designed to collect and analyse the variables that have been previously discussed to test and refine our theories. In line with the realist evaluation approach, the data collection methods employed will be comprehensive enough to capture both intended and unintended outcomes and the contextual factors and mechanisms that influenced them (46). Our objective is to audit retrospective data for a minimum period of 6 months which should allow for the thorough analysis of MSD trends, interventions, patterns, and outcomes. However, if the sample size is deemed insufficient, we will extend the data collection period beyond 6 months to ensure rich enough data and an adequate sample size for analysis.

### Qualitative data collection

Qualitative data collection methods, such as interviews and field observations, will offer valuable insights into the underlying mechanisms, contextual factors, and staff experiences that contribute to the effectiveness of DIvERT (37). By engaging with clinical staff in the selected settings and observing real-time interactions, qualitative data collection enables a deeper understanding of how DIvERT operates, how interventions are implemented, and how they impact patient outcomes. Qualitative data captures rich narratives, perspectives, and nuances that quantitative data alone may not fully capture. Qualitative data collection methods provide a comprehensive and holistic view of the complex dynamics involved, helping to uncover the “how” and “why” behind the outcomes observed, and informing recommendations for improving DIvERT (42).

### Interviews

The purpose of the theory refinement interviews is to facilitate direct engagement with clinical staff and gather firsthand accounts, thoughts, feelings, and motivations, uncovering underlying beliefs, assumptions, and values that shape their experiences and decision-making processes regarding DIvERT. Furthermore, interviews will allow participants to share their feedback, suggestions, and concerns regarding DIvERT. According to Manzano (54), the semi-structured interviews should gather the interviewees’ stories about the program because those experiences can provide insights into the program's varying processes (mechanisms, contexts, and outcomes). This teacher-learner cycle's approach should be adopted with theories placed before the respondents to comment on, confirm, deny, and refine the theories (49). An interview guide will be developed to guide the interviews. Table 4 includes examples of some of the questions we intend to ask staff to confirm, disconfirm, and refine our IPT.

**Table 4:** Examples of interview questions.

Open ended question	Can you describe a specific incident involving DIvERT and its impact on managing mental state deterioration? How do you perceive the role and effectiveness of DIvERT in addressing mental state deterioration incidents?
Probing questions	What factors do you believe contribute to the success or challenges faced by staff in assessing early warning signs of mental state deterioration? Can you share any specific examples where the response team's intervention made a significant difference in patient outcomes?
Reflective questions	Looking back, what do you think could have been done differently to improve the effectiveness of DIvERT in managing mental state deterioration presentations? How do you think DIvERT could be adapted or applied in different clinical settings?
Feedback questions	Based on your experiences, what recommendations do you have for enhancing training and preparedness of staff in managing mental state deterioration on the ward? Are there any additional resources or support systems you believe would enhance effectiveness of DIvERT?

### Who will be interviewed?

Clinical staff members participating in the DIvERT pilot program, including nurses, nurse managers, and allied health staff such as social workers, psychologist, and DIvERT team members will be interviewed. Interviews will be conducted face-to-face or virtually via the Microsoft Teams platform and recorded using a recording device. Manzano (54) suggests that realist inquiries should strive to collect a substantial amount of data. Using the purposive sampling technique, we will select participants based on their potential to contribute to refining the initial program theories (47).

For this evaluation, we anticipate conducting between 20 and 30 interviews, as suggested by M Mason (62). However, we will use saturation as our guiding principle during data collection. Saturation is achieved when additional data no longer yield new insights or information relevant to the research themes. In our study, while interviews will test predefined program theories, we will remain open to new emerging themes. We will continually code and analyse the data, closely monitoring theme development as more interviews are conducted. Regular reviews of themes and codes will be performed to ensure both depth and breadth in the data collected. Saturation will be considered reached once the data begin to repeat, with no new information contributing to the data collection process.

### Field observations

Observational research methods are essential for understanding people's actions, roles and behaviours in their natural environments (63). Field observations will involve gathering rich contextual data that complements interviews, surveys, and document analysis. The field observations are aimed to understand the “what is going on” phenomena (64, 65). The researcher will attend DIvERT activations on the medical wards as an observer to see how DIvERT functions in real time and to note the interaction patterns among healthcare professionals, including how they communicate and coordinate to meet MSD needs. Additionally, we will track the timeliness of responses, from the initial call to the team's arrival and their subsequent interventions. We will also monitor immediate patient outcomes post-DIvERT to observe any changes in the patient's mental state. Furthermore, we will identify challenges and barriers that impact the effective functioning of DIvERT.

The researcher will take field notes to document the events and activities that occur during the response calls. The data gathered through field observations can provide valuable insights for refining the DIvERT model, identifying areas for improvement, and informing recommendations for enhancing the management of patient MSD incidents. We aim to complete sufficient, but not exhaustive, field observations to gather data to test and refine the theories. We will follow the principles of reasonable judgment and determine the point of reaching contextual richness (51). Once it is determined that attending additional field observations will not yield new information, we will conclude the field observations.

### Step 4: configuring collected data about initial theories: data synthesis

In realist methodology, the purpose of data analysis is to uncover the underlying mechanisms and contextual factors that influence program outcomes through a confirmatory theory building theory-building approach (42, 46). To draw inferences from the evidence, the primary approach of constructing confirmatory theories, particularly within realist research, is retroductive reasoning (or abductive reasoning) (66). Analysis involves cross-examining program theories with collected data and using theories to understand configurations in data (34). Data analysis is aimed not merely to summarise and report themes in the data, nor is the approach designed to aggregate data by strength in numbers philosophy. Rather, it involves data analysis to improve an ontologically deep understanding of the phenomena being studied (38).

### Quantitative data analysis

Microsoft Excel and STATA (Statistics and Data) will be used to process, organise, and analyse quantitative data. The main objectives of the quantitative data analysis are to:

1. Summarise and present data using descriptive statistics and display data as frequency tables, and diagrams.
2. Test if there are differences in Code Grey responses before and after DIvERT intervention implementation.
3. Analyse patient outcomes associated with mental state deterioration.

### Qualitative data analysis

The qualitative data will be managed using NVivo. NVivo is a software program used for qualitative and mixed-methods research. NVivo helps qualitative researchers to organise, analyse and find insights in unstructured or qualitative data where deep levels of analysis are required (67). For realist approaches, NVivo offers a framework to structure and organise the iterative process of generating, refining, and testing complex program theories, especially when drawing on multiple data sources simultaneously (68). This is particularly beneficial given the multifaceted and dynamic nature of realist data (68). Qualitative data will be transcribed, organised, and labelled using Microsoft Word.

There are different ways of coding and reporting data, holistic coding, referential coding, and axial coding (38). For holistic coding, a single code is applied to a large unit of data and is most applicable when the researcher has a general idea of what to investigate in the data. In referential coding, researchers identify and code specific phrases in the data, focusing on instances where participants explicitly or implicitly refer to certain concepts, themes, and ideas. Thirdly, axial coding involves the breakdown and categorisation of data into specific themes, concepts, or categories based on their relationships (69). By exploring Context-Mechanism-Outcome relationships allows for a more in-depth analysis of how various elements interrelate and enables the identification of patterns that contribute to improved conceptual clarity. This evaluation will be based on the latter approach.

Transcribing and coding will be piloted following a few interviews to clarify the approach. The analytical process in realist methodology is iterative, meaning that coding and analysis can be started as data are collected (38). For interview, data analysis will be based on thematic analysis and survey data, we will adopt utilise content analysis. Using data to support, refute, and refine program theories, we will primarily focus on extrapolating the lines of association (46). The results will support inferences about varied influences operating within a specific CMO configurations. Labelled CMO configurations will attempt to explain how and why various mechanisms are activated or not in different contexts to generate different outcomes (34). CMO configurations are about specific characteristics of the program, and when considered as a whole, they become the product of the complete review (70). As new theories supporting causal insights emerge during data analysis, they will be articulated and labelled accordingly. Regular fortnightly meetings with academic supervisors will be held throughout the research process to check and validate analysis process, review overall progress, and address challenges.

### Step 5: present the research output

*(Present configured contexts and mechanisms into refined program theories)*.

In this phase the refined and evidence-informed theories to support “what works, for whom, under what circumstances, how and what did not work” about the DIvERT intervention will be presented (42). Refined theories will be presented with corresponding CMO configurations. Refined theories will guide future DIvERT program design and implementation. As pointed out, propositions for policy and practice should be explained clearly, follow logically from the analysis, and recommendations should account for strategies for different contexts (46). It is prudent for realist evaluations to be modest about evaluation claims, as evaluation results cannot be universally applicable. Rather, results should provide plausible explanations for the complex processes underlying programs (42).

We will engage with key stakeholders in the final stages of the realist evaluation, an important step that serves dual objectives. Firstly, engagement provides a means to validate the research findings, aligning them with the practical experiences and perspectives of those directly involved. This validation not only improves the credibility of the evaluation but also ensures that the results align with the stakeholders’ intentions of DIvERT. Secondly, engaging stakeholders facilitates a dynamic exchange of insights, enriching the understanding of contextual nuances that may influence the success of DIvERT. The collaborative process improves the applicability of the evaluation's recommendations, fostering stakeholder buy-in and promoting the effective implementation of evidence-based strategies in other clinical settings. As per reporting standards, strengths and limitations of the methodology, utility, and analysis will be reported (46).

## Conclusion

Currently, there is a lack of consistency in the early identification and management of patient MSD in acute hospital settings. In particular, the effectiveness of interventions in this area is not well understood. Furthermore, as reported staff feel unsafe, unsupported, and often under-skilled to manage patient MSD in acute hospital settings, which negatively impacts patient care and outcomes. The DIvERT intervention is a proactive model of care designed to initiate interventions at the first signs of deterioration. This is an effort to support the patient's journey by ensuring a timely appropriate plan of care and support clinical ward teams to identify and manage patient MSD proactively. This evaluation will help fill the knowledge gap on the effectiveness of interventions for managing patient mental state deterioration in acute medical settings. Additionally, findings from this research will offer opportunities to improve the clinical skills of staff through tailored training programs to assess, manage and escalate care for patients presenting with MSD effectively and safely. DIvERT is part of a comprehensive organisational initiative to identify and implement innovative strategies to minimise patient MSD, enhancing staff capability and resilience. In ending, in a continuously evolving health care environment, it is imperative to promptly incorporate new evidence-based interventions into clinical practice. This urgency is driven by the need to keep pace with evolving challenges and advancements, ensuring that patient care is continually improved. In trialling DIvERT, we trying to learn from the proposed realist evaluation to ensure that the rapid translation of the program not only optimises outcomes for our patients but also maximises impact at the organisational, service, and system levels. As a result of this flexibility in implementation, resources can be allocated more effectively, resulting in sustained improvements and adaptability within the broader healthcare framework.

## References

[1] National Consensus Statement: Essential elements for recognising and responding to deterioration in a person's mental state. Available online at: https://www.safetyandquality.gov.au/sites/default/files/migrated/National-Consensus-Statement-Essential-elements-for-recognising-and-responding-to-deterioration-in-a-person%E2%80%99s-mental-state-July-2017.pdf (Accessed October 23, 2023).

[2] Lamont S Brunero S. The effect of a workplace violence training program for generalist nurses in the acute hospital setting: a quasi-experimental study. Nurse Educ Today. (2018) 68:45–52. 10.1016/j.nedt.2018.05.008 29885569

[3] Morphet J Griffiths D Beattie J Velasquez Reyes D Innes K. Prevention and management of occupational violence and aggression in healthcare: a scoping review. Collegian. (2018) 25(6):621–32. 10.1016/j.colegn.2018.04.003

[4] Violence and aggression: short-term management in mental health, health and community settings. Available online at: https://www.nice.org.uk/guidance/ng10/resources/violence-and-aggression-shortterm-management-in-mental-health-health-and-community-settings-pdf-1837264712389 (Accessed October 10, 2023).

[5] Arnetz JE Hamblin L Russell J Upfal MJ Luborsky M Janisse J Preventing patient-to-worker violence in hospitals: outcome of a randomized controlled intervention. J Occup Environ Med. (2017) 59(1):18–27. 10.1097/JOM.0000000000000909 28045793 PMC5214512

[6] Gaskin C. Recognising signs of deterioration in a person’s mental state: an updated literature review. (2019). Available online at: https://www.safetyandquality.gov.au/sites/default/files/migrated/Recognising-Signs-of-Deterioration-in-a-Persons-Mental-State-Gaskin-Research-Final-Report.pdf (Accessed September 19, 2023).

[7] Mental Health and Wellbeing Act 2022 no. 39 of 2022. Available online at: https://content.legislation.vic.gov.au/sites/default/files/2022-09/22-039aa%20authorised.pdf (Accessed September 12, 2023).

[8] Department of Health. Physical Restraint Standardised Care Process. Melbourne: Victoria State Government (2022). Available online at: https://www.health.vic.gov.au/sites/default/files/2022-12/standardise-care-physical-restraint.PDF (Accessed September 12, 2023).

[9] Muir-Cochrane E Muller A Fu Y Oster C. Role of security guards in code black events in medical and surgical settings: a retrospective chart audit. Nurs Health Sci. (2020) 22(3):758–68. 10.1111/nhs.12725 32314506

[10] Maguire T Daffern M Bowe SJ McKenna B. Predicting aggressive behaviour in acute forensic mental health units: a re-examination of the dynamic appraisal of situational aggression’s predictive validity. Int J Ment Health Nurs. (2017) 26(5):472–81. 10.1111/inm.12377 28960740

[11] Recognising Signs of Deterioration in a Person’s Mental State: An Updated Literature Review. Available online at: https://www.safetyandquality.gov.au/sites/default/files/2019-11/recognising_signs_of_deterioration_in_a_persons_mental_state_an_updated_literature_review_0.pdf (Accessed October 5, 2023).

[12] Semple D Smyth R. Oxford Handbook of Psychiatry. Oxford: Oxford University Press (2019).

[13] Mento C Silvestri MC Bruno A Muscatello MRA Cedro C Pandolfo G Workplace violence against healthcare professionals: a systematic review. Aggress Violent Behav. (2020) 51:101381. 10.1016/j.avb.2020.101381

[14] Prevention and management of violence and aggression in health services. Available online at: https://content.api.worksafe.vic.gov.au/sites/default/files/2018-06/ISBN-Prevention-and-management-of-violence-and-aggression-health-services-2017-06.pdf (Accessed October 5, 2023).

[15] Nambiar D Pearce JW Bray J Stephenson M Nehme Z Masters S Variations in the care of agitated patients in Australia and New Zealand ambulance services. Emerg Med Australas. (2020) 32(3):438–45. 10.1111/1742-6723.13431 31840407

[16] Parsch CS Boonstra A Teubner D Emmerton W McKenny B Ellis DY. Ketamine reduces the need for intubation in patients with acute severe mental illness and agitation requiring transport to definitive care: an observational study. Emerg Med Australas. (2017) 29(3):291–6. 10.1111/1742-6723.12763 28320079

[17] Occupational violence against healthcare workers. Available online at: https://www.audit.vic.gov.au/sites/default/files/2017-07/20150506-Occ-Violence.pdf? (Accessed October 23, 2023).

[18] Li Y-L Li R-Q Qiu D Xiao S-Y. Prevalence of workplace physical violence against health care professionals by patients and visitors: a systematic review and meta-analysis. Int J Environ Res Public Health. (2020) 17(1):299. 10.3390/ijerph17010299 31906306 PMC6982349

[19] Cole R. Reducing restraint use in a trauma center emergency room. Nurs Clin North Am. (2014) 49(3):371–81. 10.1016/j.cnur.2014.05.010 25155536

[20] Iozzino L Ferrari C Large M Nielssen O De Girolamo G. Prevalence and risk factors of violence by psychiatric acute inpatients: a systematic review and meta-analysis. PLoS One. (2015) 10(6):e0128536. 10.1371/journal.pone.0128536 26061796 PMC4464653

[21] Laschinger HKS Grau AL. The influence of personal dispositional factors and organizational resources on workplace violence, burnout, and health outcomes in new graduate nurses: a cross-sectional study. Int J Nurs Stud. (2012) 49(3):282–91. 10.1016/j.ijnurstu.2011.09.004 21978860

[22] Lee J Daffern M Ogloff JR Martin T. Towards a model for understanding the development of post-traumatic stress and general distress in mental health nurses. Int J Ment Health Nurs. (2015) 24(1):49–58. 10.1111/inm.12097 25279764

[23] Lanctôt N Guay S. The aftermath of workplace violence among healthcare workers: a systematic literature review of the consequences. Aggress Violent Behav. (2014) 19(5):492–501. 10.1016/j.avb.2014.07.010

[24] Royal College of Nursing. Violence and Aggression in the NHS: Estimating the Size and the Impact of the Problem–an interim Report London: Royal College of Nursing (2018).

[25] Buchell H Whitecross F Berry C Sonmez G Moran J Rauchberger I Exploring the Prevalence and Impact of Behaviours of Concern and Whether a Psychiatric Behaviour of Concern Team Improves Safety. Alfred Health. (2018) Available online at: https://healthsciences.unimelb.edu.au/__data/assets/pdf_file/0009/2857527/Fiona-Whitecross-exploring-the-impact-and-prevalence-of-behaviours.pdf (Accessed October 23, 2023).

[26] Kaunomäki J Jokela M Kontio R Laiho T Sailas E Lindberg N. Interventions following a high violence risk assessment score: a naturalistic study on a Finnish psychiatric admission ward. BMC Health Serv Res. (2017) 17(1):26. 10.1186/s12913-016-1942-0 28077156 PMC5225613

[27] Ogloff JR Daffern M. The dynamic appraisal of situational aggression: an instrument to assess risk for imminent aggression in psychiatric inpatients. Behav Sci Law. (2006) 24(6):799–813. 10.1002/bsl.741 17171770

[28] Dumais A Larue C Michaud C Goulet M-H. Predictive validity and psychiatric nursing staff’s perception of the clinical usefulness of the French version of the dynamic appraisal of situational aggression. Issues Ment Health Nurs. (2012) 33(10):670–5. 10.3109/01612840.2012.697254 23017043

[29] Code Grey Standards. Available online at: https://www2.health.vic.gov.au/about/publications/policiesandguidelines/code-grey-standards (Accessed October 4, 2023).

[30] Weapons management in Victorian health services. Available online at: https://www.health.vic.gov.au/publications/weapons-management-in-victorian-health-services (Accessed October 4, 2023).

[31] Nia Manning S. Managing behaviour that challenges in people with dementia in the emergency department. Emerg Nurse. (2021):34–40. 10.7748/en.2020.e2019 33377357

[32] Edward K-L Giandinoto J-A Weiland TJ Hutton J Reel S. Brief interventions to de-escalate disturbances in emergency departments. Br J Nurs. (2018) 27(6):322–7. 10.12968/bjon.2018.27.6.322 29561674

[33] Digby R Bushell H Bucknall TK. Implementing a psychiatric behaviours of concern emergency team in an acute inpatient psychiatry unit: staff perspectives. Int J Ment Health Nurs. (2020) 29(5):888–98. 10.1111/inm.12723 32243059

[34] Wong G Westhorp G Greenhalgh J Manzano A Jagosh J Greenhalgh T. Quality and reporting standards, resources, training materials and information for realist evaluation: the RAMESES II project. Health Serv Deliv Res. (2017) 5(28):1–108. 10.3310/hsdr05280 29072890

[35] Jagosh J. Realist synthesis for public health: building an ontologically deep understanding of how programs work, for whom, and in which contexts. Annu Rev Public Health. (2019) 40(1):361–72. 10.1146/annurev-publhealth-031816-044451 30633712

[36] Marchal B Van Belle S Van Olmen J Hoerée T Kegels G. Is realist evaluation keeping its promise? A review of published empirical studies in the field of health systems research. Evaluation. (2012) 18(2):192–212. 10.1177/1356389012442444

[37] Pawson R. Evidence-Based Policy: A Realist Perspective. London: Sage (2006).

[38] Jagosh J. 8th annual CARES summer school for realist methodology training. (2021).

[39] Pawson R. Theorizing the interview. Br J Sociol. (1996) 47(2):295. 10.2307/591728

[40] Park YS Konge L Artino AR Jr. The positivism paradigm of research. Acad Med. (2020) 95(5):690–4. 10.1097/ACM.0000000000003093 31789841

[41] Adom D Yeboah A Ankrah AK. Constructivism philosophical paradigm: implication for research, teaching and learning. Glob J Arts Humanit Soc Sci. (2016) 4(10):1–9. Available online at: https://www.eajournals.org/wp-content/uploads/Constructivism-Philosophical-Paradigm-Implication-for-Research-Teaching-and-Learning.pdf

[42] Pawson R Tilley N. Realistic Evaluation. London: Sage (1997).

[43] Bhaskar R. A Realist Theory Of Science. Oxford: Routledge (2013).

[44] Williams L Rycroft-Malone J Burton CR. Bringing critical realism to nursing practice: Roy Baskar’s contribution. Nurs Philos. (2017) 18(2):e12130. 10.1111/nup.12130 27381640

[45] Sayer A. Realism and Social Science. London: SAGE Publications Ltd. (2000).

[46] Wong G Westhorp G Manzano A Greenhalgh J Jagosh J Greenhalgh T. RAMESES II reporting standards for realist evaluations. BMC Med. (2016) 14(1):96. 10.1186/s12916-016-0643-1 27342217 PMC4920991

[47] Mukumbang FC Marchal B Van Belle S Van Wyk B. Unearthing how, why, for whom and under what health system conditions the antiretroviral treatment adherence club intervention in South Africa works: a realist theory refining approach. BMC Health Serv Res. (2018) 18(1):1–15. 10.1186/s12913-018-3150-6 29743067 PMC5944119

[48] Realist impact evaluation: an introduction. Available online at: https://cdn.odi.org/media/documents/9138.pdf (Accessed September 18, 2023).

[49] Mukumbang FC Marchal B Van Belle S Van Wyk B. Using the realist interview approach to maintain theoretical awareness in realist studies. Qual Res. (2020) 20(4):485–515. 10.1177/1468794119881985

[50] Pawson R. Digging for nuggets: how ‘bad’ research can yield ‘good’ evidence. Int J Soc Res Methodol. (2006) 9(2):127–42. 10.1080/13645570600595314

[51] Wong G Greenhalgh T Westhorp G Buckingham J Pawson R. RAMESES Publication standards: realist syntheses. BMC Med. (2013) 11(1):21. 10.1186/1741-7015-11-21 23360677 PMC3558331

[52] Pawson R Greenhalgh T Harvey G Walshe K. Realist review—a new method of systematic review designed for complex policy interventions. J Health Serv Res Policy. (2005) 10(1_suppl):21–34. 10.1258/1355819054308530 16053581

[53] Pawson R. Evidence-based policy: the promise of `realist synthesis. Evaluation. (2002) 8(3):340–58. 10.1177/135638902401462448

[54] Manzano A. The craft of interviewing in realist evaluation. Evaluation. (2016) 22(3):342–60. 10.1177/1356389016638615

[55] Pawson R. The Science of Evaluation: A Realist Manifesto. London: Sage Publications (2013).

[56] Creswell JW Creswell JD. Research Design: Qualitative, Quantitative, and Mixed Methods Approaches. London: Sage Publications (2017).

[57] National safety and quality health service standards. Available online at: https://www.safetyandquality.gov.au/sites/default/files/2021-05/national_safety_and_quality_health_service_nsqhs_standards_second_edition_-_updated_may_2021.pdf (Accessed September 18, 2023).

[58] Safer Care Victoria. Caring for people displaying acute behavioural disturbance: clinical guidance to improve care in emergency settings. (2020). Available online at: https://www.safercare.vic.gov.au/best-practice-improvement/clinical-guidance/emergency/acute-behavioural-disturbance (Accessed September 18, 2023).

[59] Mukumbang FC. Retroductive theorizing: a contribution of critical realism to mixed methods research. J Mix Methods Res. (2023) 17(1):93–114. 10.1177/15586898211049847

[60] Brtnikova M Crane LA Allison MA Hurley LP Beaty BL Kempe A. A method for achieving high response rates in national surveys of U.S. primary care physicians. PLoS One. (2018) 13(8):e0202755. 10.1371/journal.pone.0202755 30138406 PMC6107210

[61] Aday LA Cornelius LJ. Designing and Conducting Health Surveys: A Comprehensive Guide. John Wiley & Sons (2006).

[62] Mason M. Sample size and saturation in PhD studies using qualitative interviews. Forum Qual Soz Forsch. (2010) 11(3). 10.17169/fqs-11.3.1428

[63] Walshe C Ewing G Griffiths J. Using observation as a data collection method to help understand patient and professional roles and actions in palliative care settings. Palliat Med. (2012) 26(8):1048–54. 10.1177/0269216311432897 22179595

[64] Morgan SJ Pullon SRH Macdonald LM McKinlay EM Gray BV. Case study observational research: a framework for conducting case study research where observation data are the focus. Qual Health Res. (2017) 27(7):1060–8. 10.1177/1049732316649160 27217290

[65] Adams J. Assessing the effectiveness of clinical education to reduce the frequency and recurrence of workplace violence. Aust J Adv Nurs. (2017) 34(3):6–15. 10.37464/2017.343.1520

[66] Eastwood JG Jalaludin BB Kemp LA. Realist explanatory theory building method for social epidemiology: a protocol for a mixed method multilevel study of neighbourhood context and postnatal depression. SpringerPlus. (2014) 3(1):12. 10.1186/2193-1801-3-12 24422187 PMC3888492

[67] Bergeron DA Gaboury I. Challenges related to the analytical process in realist evaluation and latest developments on the use of NVivo from a realist perspective. Int J Soc Res Methodol. (2020) 23(3):355–65. 10.1080/13645579.2019.1697167

[68] Dalkin S Forster N Hodgson P Lhussier M Carr SM. Using computer assisted qualitative data analysis software (CAQDAS; NVivo) to assist in the complex process of realist theory generation, refinement and testing. Int J Soc Res Methodol. (2021) 24(1):123–34. 10.1080/13645579.2020.1803528

[69] Saldaña J. The Coding Manual for Qualitative Researchers. London: Sage (2021).

[70] Jagosh J Bush PL Salsberg J Macaulay AC Greenhalgh T Wong G A realist evaluation of community-based participatory research: partnership synergy, trust building and related ripple effects. BMC Public Health. (2015) 15(1):1–11. 10.1186/s12889-015-1949-1 26223523 PMC4520009

